# “The Shop is Not Closed”: Sex and Sexuality Among Older Adult Daters

**DOI:** 10.1093/geroni/igaf122.3695

**Published:** 2025-12-31

**Authors:** Lauren Harris, Celia Melanson

**Affiliations:** University of New Hampshire, Durham, New Hampshire, United States; University of New Hampshire, Durham, New Hampshire, United States

## Abstract

There is a common myth that older adults are not sexual people, either from a lack of desire or ability. This study focuses on the experiences of single older adults on the dating market to understand the role of sexual desire and expectation for a population often assumed to be uninterested in or incapable of sexual activity. Previous research has largely focused on younger or partnered individuals, leaving the experiences of single older adults under-explored. Through this study, we seek to understand the desires, preferences, and challenges faced by single older adults who are seeking romantic and sexual relationships. It investigates how factors such as sexual dysfunction, stereotypes about aging bodies, and limited opportunities for finding sexual partners impact their willingness to pursue and their expectations of intimacy. Results indicate that, despite physical and emotional challenges related to aging, sexual activity remains an important aspect of romantic relationships for many single older adults. Both men and women recognize the impact of aging on sexual function but do not view these changes as insurmountable barriers. Rather, they express a desire for intimacy and adjust their expectations to accommodate physical changes. The study challenges agist stereotypes and highlights the need to normalize sexual desire in later life. The findings contribute to a broader understanding of older adult sexuality, particularly for those navigating the dating market, and provide insight into how aging adults perceive and prioritize sexual intimacy in relationships.

